# Measles Outbreak Response Immunization Is Context-Specific: Insight from the Recent Experience of Médecins Sans Frontières

**DOI:** 10.1371/journal.pmed.1001544

**Published:** 2013-11-05

**Authors:** Andrea Minetti, Cameron Bopp, Florence Fermon, Gwenola François, Rebecca F. Grais, Lise Grout, Northan Hurtado, Francisco J. Luquero, Klaudia Porten, Laurent Sury, Meguerditch Terzian

**Affiliations:** 1 Epicentre, Paris, France; 2 Médecins Sans Frontières, Paris, France

## Abstract

Andrea Minetti and colleagues compare measles outbreak responses from the Democratic Republic of the Congo and Malawi and argue that outbreak response strategies should be tailored to local measles epidemiology.

*Please see later in the article for the Editors' Summary*

**Summary Points**

- During the recent resurgence of measles in sub-Saharan Africa, the majority of cases were reported from the Democratic Republic of the Congo and Malawi, two countries with vastly different measles epidemiology.
- Non-selective mass vaccination campaigns targeting children aged 6 months to <15 years old are the commonly implemented strategy for responding to measles outbreaks in humanitarian emergencies.
- Differences in measles epidemiology and country-specific control goals necessitate more than a one-size-fits-all strategy.
- Measles outbreak responses should be tailored to local measles epidemiology following early assessment: the age distribution of early cases should guide the decision on which age groups to vaccinate.
- In settings where the main objective is mortality reduction, the youngest children—who account for the most deaths and complications—should be prioritized by the outbreak response.

In 2003, the World Health Assembly endorsed a resolution to reduce measles deaths by 50% compared with 1999 estimates by the end of 2005 [1]. This target was met [2],[3], and a new goal was established to achieve 90% death reduction by 2010, compared with mortality in 2000 [4],[5]. This new objective has not been reached [6], jeopardizing the World Health Organization (WHO) goal of measles elimination in the WHO African region by 2020 [7]. An elimination goal has been pursued by some countries in southern Africa since 1996 [8]. Despite undeniable achievements, a recent resurgence of measles highlights the challenge of sustaining elimination goals and the uneven progress across sub-Saharan Africa [9],[10]. In 2010–2011, several sub-Saharan African countries experienced measles outbreaks, with more than 199,000 cases officially reported to WHO in 2010, and more than 194,000 in 2011 [11]. These recent, large measles epidemics in Africa, especially in countries with a history of successful measles control, are of great concern [12].

## Current Practice for Measles Outbreak Response

WHO guidelines for outbreak response immunization (ORI) recommend considering measles control goals, background vaccination coverage, age distribution of cases, and case fatality rates when planning measles vaccination. Results from the outbreak investigation and prior surveillance data should be used to develop and tailor an appropriate response. When preliminary investigation indicates a high risk of a large outbreak, a non-selective mass vaccination campaign should be implemented, and all age groups contributing to cases should be targeted, to avert the largest number of cases and to decrease transmission [13]. Unfortunately, in the field, these guidelines are seldom applied or are poorly implemented. In common practice, at best, measles vaccination interventions follow the Sphere Project recommendation for humanitarian emergencies: vaccinating against measles in all children between the ages 6 months and <15 years old [14]. The same target age range is generally recommended for outbreak response interventions in order to optimize impact [15]. However, differences in measles epidemiology and control goals necessitate more than a one-size–fits-all strategy. The public health objectives of a measles outbreak response are context-specific, depending essentially upon routine vaccination and the performance of supplementary immunization activities (SIAs). The age distribution of measles cases varies across sub-Saharan African countries, reflecting different levels of preexisting population immunity. This variation is a consequence of past and ongoing control programs and the degree of circulating virus [16]. Whether the goal in the country is mortality reduction or measles elimination is a critical element to be considered when planning and implementing ORI.

## Two Measles Epidemics with Different Epidemiological Profiles

During the measles resurgence in 2010–2011, the majority of cases in the African region were reported from the Democratic Republic of the Congo (DRC) and Malawi, two countries with vastly different measles epidemiology. Non-selective mass vaccination campaigns targeting children aged 6 months to <15 years old were a part of the Médecins Sans Frontières (MSF) outbreak response strategy in response to these epidemics. In both countries, as part of the support to the countries' ministries of health, MSF strengthened the surveillance system in health zones or districts where ORI was implemented. This process included reinforcement of health officer training in case definition and data collection, retrospective review of health registers, weekly communication of data to the district level, monitoring of data completeness, and electronic data compilation. In both countries, national-level surveillance is based on the Integrated Disease Surveillance and Response strategy, and WHO definitions for measles cases and deaths are used. Once the outbreak is laboratory-confirmed, additional cases are confirmed clinically [13]. Therefore, it is possible that some of the measles cases are misclassified as rubella.

Here, we provide an overview of the different epidemiology of these two epidemics in terms of the age distribution of cases and discuss the need to reinforce context-specific strategies. In DRC, between week 23 of 2010 and week 52 of 2011, 128,113 measles cases and 1,454 deaths were reported, with the bulk of reported cases (60%) from Katanga Province. In Katanga, the overall cumulative attack rate (AR) was 0.71%, and the case fatality ratio (CFR) was 1.40%. In Malawi, between week 1 of 2010 and week 52 of 2010, 134,039 measles cases and 304 deaths were reported, with a cumulative AR of 0.96% and a CFR of 0.23%.

**Figure 1:**
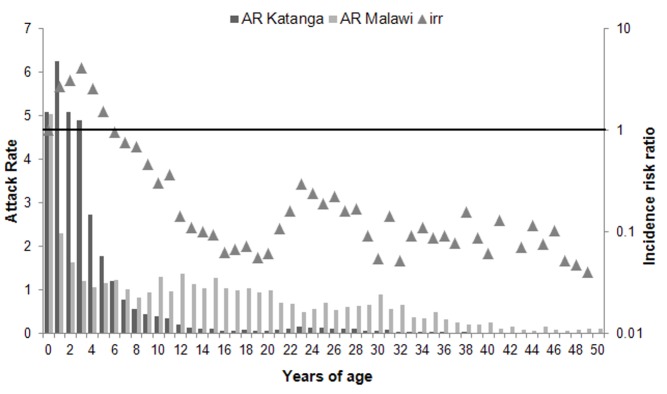
Age distribution of measles cases in Katanga Province (Democratic Republic of the Congo), 2010–2011, and in Malawi, 2010, as represented by attack rates, with incidence risk ratio by age. The incidence risk ratio (irr) is the age-specific AR in Katanga divided by the age-specific AR in Malawi.

The epidemiological profiles of these two epidemics are distinguishable by the age distribution of cases. In Katanga, the median age of cases was 2 years (interquartile range: 1–4), with 80% of reported cases in children <5 years old and only 6% in individuals ≥10 years old. In Malawi, the median age of cases was 7 years (interquartile range: 1–16), with 41% of reported cases in children <5 years old and 28% in individuals ≥15 years old (Table 1). In both countries, almost a fifth of cases were in children <1 years old—including children in the Expanded Programme on Immunization target group and younger—with ARs around 5%. In Katanga, young children (12–59 months old) were also highly affected, with AR sharply decreasing in older children. In Malawi, the AR was lower for young children than for children of the same age in Katanga, but much higher for age groups >;5 years old, as shown by the incidence risk ratio (Figure 1).

**Table 1:** Measles cases, deaths, attack rates , and case fatality ratios by age group, reported in Malawi between week 1 and week 52 of 2010 and in Katanga Province between week 23 of 2010 and week 52 of 2011.

Age Group	Malawi				Katanga Province (DRC)^a^			
	Number of Cases (Percent)	AR per 100	Number of Deaths (Percent)	CFR per 100	Number of Cases (Percent)	AR per 100	Number of Deaths (Percent)	CFR per 100
All	134,039	0.96	304	0.23	45,356	1.17	197	0.43
0–5 months	7,243 (5%)	2.26	10 (3%)	0.14	1,851 (4%)	2.31	7 (4%)	0.38
6–8 months	10,615 (8%)	7.61	27 (9%)	0.25	3,395 (7%)	8.49	22 (11%)	0.65
9–11 months	7,543 (6%)	4.50	21 (7%)	0.28	2,962 (7%)	7.40	19 (10%)	0.64
12–59 months	28,737 (22%)	1.38	81 (28%)	0.28	28,098 (62%)	4.68	126 (64%)	0.45
5–9 years	20,434 (16%)	1.05	44 (15%)	0.21	6,228 (14%)	0.97	17 (9%)	0.27
10–14 years	19,545 (15%)	1.00	25 (9%)	0.13	1,293 (3%)	0.23	3 (2%)	0.23
15–19 years	13,641 (10%)	1.00	14 (5%)	0.10	287 (1%)	0.08	0	0
≥20 years	23,965 (18%)	0.40	70 (24%)	0.29	847 (2%)	0.05	2 (1%)	0.24

Percent values in parentheses are percent of all cases or deaths that are in that age group. Population figures for Malawi are a projection from estimates of the 2008 Population and Housing Census [24]; population figures for Katanga are a projection from estimates of the 2007 Demographic and Health Survey [25].

a Data are from only the 28 health zones of Katanga where surveillance was reinforced.

## Outbreak Response in Katanga, a Post-Conflict Setting

In post-conflict countries, with long-term disruption of immunization programs and poor access to health care, measles is a major cause of child mortality and further exacerbates subjacent malnutrition. In such contexts, the identification of groups at higher risk of dying from measles is essential to guide resource allocation for outbreak response. In DRC, measles transmission is high, epidemics are recurrent, and, typically, young children are by far the most affected. Previous studies highlight that measles CFR is higher in unvaccinated children <5 years old than in older children, with a decreasing trend with each year increase in age [17]. Unacceptably high measles-related mortality has been documented in children, particularly in countries in sub-Saharan Africa that have failed to implement the WHO-recommended strategy for sustainable measles mortality reduction. In such contexts, age-specific measles CFRs show that infants and children <3 years old are at highest risk of death [18]. The main objective of ORI in these settings should be mortality reduction. Deaths and measles complications can be reduced by ensuring appropriate free treatment of cases and protection through immunization. When resources are limited (insufficient vaccine supplies, lack of trained staff, limited logistical capacity) and the timely access to large affected populations is needed, younger age groups should be considered a priority and should be vaccinated as soon as possible, with the objective of reducing severe complications and mortality [15].

During the outbreak in Katanga, MSF vaccinated over 2.1 million individuals aged 6 months to <15 years in 26 health zones. In the Haut-Lomami district of Katanga, the target age group was restricted to children aged 6 months to <10 years, with 252,559 individuals vaccinated in four health zones [19]. The decision to limit the target age group was based on the age distribution of early cases, showing low AR among individuals ≥10 years old, to optimize the use of available resources. Although not the strategy implemented, a more efficient use of limited resources and a greater coverage of young children at highest risk of death could have been achieved by further limiting the target age group.

## Outbreak Response in Malawi, a Stable Country

In stable countries with a measles elimination goal, major outbreaks should be avoided through a combination of good routine coverage and consistent, high-quality SIAs. Outbreaks in these settings are the result of gaps in the routine immunization program or failures of SIAs to catch up non-immunized children. When an outbreak is declared in such contexts, all age groups contributing to cases should be targeted during the measles outbreak response, to avert the largest number of cases and to decrease transmission. This should be possible where and if enough resources are allocated to measles control.

Despite high reported measles vaccine coverage and sustained low measles transmission, in 2010 Malawi faced its largest measles outbreak in more than two decades. During the 2010 epidemic, age groups ≥15 years contributed almost one-third of the cases, as expected in a setting with good, but not sustained, vaccine coverage and effectiveness [10]. During the Malawi epidemic, MSF vaccinated through non-selective mass campaigns over 3.3 million children aged 6 months to <15 years, half of the country's total population for this age group. However, outbreak response efforts failed to control the epidemic because vaccination campaigns were implemented late in the course of the epidemic, and transmission was sustained by older individuals not targeted by the ORI campaign. Targeting individuals ≥15 years old would have had a greater impact on the transmission dynamics than did limiting vaccination to those <15 years old. However, the financial, human resource, and logistical costs of such interventions are substantial and should be taken into account during planning. Finally, the choice between width of target age range and geographical coverage should always consider a reasonable balance between feasibility, cost, and expected impact.

## The Need for a Context-Specific Approach

In both settings, the highest ARs occurred among infants 6–8 months old, too young to be vaccinated by routine programs. The benefit of vaccinating earlier in regions with high birth rates, as in Katanga, has been previously discussed [20]. In Malawi, young infants aged 0–5 months were highly affected, with an AR higher than that of children 12 months and older. Recent studies showed lower concentrations and earlier loss of maternal antibodies against measles in infants of vaccinated women than in infants of naturally immune women [21]. In countries with effective measles vaccination programs, maternal antibody levels against measles may therefore be low. In such contexts, early vaccination of infants should be considered in the case of an outbreak, as described elsewhere [22].

In both settings, ARs were higher for young children born after the last SIA. Among these children, the proportion receiving their first or second dose during the MSF reactive campaign was high, suggesting that non-selective strategies are efficient for poorly vaccinated age groups or those that have not been offered their second immunization opportunity. Nonetheless, the high proportion of older individuals reporting having received more than two doses suggests that non-selective vaccination might not be the most cost-effective option for highly vaccinated populations. In such contexts, cost–benefit studies are urgently needed to evaluate the differential benefit in terms of disease prevention and mortality reduction [23] of continuing with non-selective strategies to deliver the second dose, whether in SIAs or ORI campaigns.

As measles control improves across sub-Saharan Africa, outbreak response vaccination should also keep pace with these improvements. Countries should set their own priorities for outbreak response, targeted to their measles control goals. Immunization strategies should be tailored to local measles epidemiology, following early assessment. In particular, the age distribution of early cases should guide the decisions concerning which age groups to target in priority. Measles vaccination is an essential component of outbreak response in settings where the main objective is mortality reduction. In those contexts, the youngest children—accounting for the most deaths and complications—should be prioritized.

## Abbreviations

AR: attack rate
CFR: case fatality ratio
DRC: Democratic Republic of the Congo
MSF: Médecins Sans Frontières
ORI: outbreak response immunization
SIA: supplementary immunization activity
WHO: World Health Organization
